# Incidental Clear Cell Syringoma of the Scalp in a Patient With Lichen Planopilaris

**DOI:** 10.7759/cureus.16064

**Published:** 2021-06-30

**Authors:** Jihee Choi, Jaime Tschen, Philip R Cohen

**Affiliations:** 1 Dermatology, St. Joseph Dermatopathology, Houston, USA; 2 Dermatology, San Diego Family Dermatology, National City, USA

**Keywords:** alopecia, cell, clear, lichen, non-scarring, planopilaris, planus, scalp, scarring, syringoma

## Abstract

Syringomas are benign neoplasms of eccrine ducts; glycogen accumulation in the tumor cell cytoplasm results in a clear cell variant of syringoma. Syringoma and syringomatous proliferations (secondary to alteration of the eccrine sweat ducts) have been observed, albeit uncommonly, as an incidental finding in areas of alopecia on the scalp. A 71-year-old woman with scalp hair loss caused by lichen planopilaris had subclinical clear cell syringoma discovered as an incidental observation on evaluation of the biopsy specimen from an area of hair loss. Including our patient, scalp alopecia-associated syringoma or syringomatous proliferation has been described in a 47-year-old man and 16 women. The women ranged in age from 33 years to 83 years (median, 57 years). The duration of alopecia ranged from six months to 22 years; almost half of the patients (three of seven) had hair loss for 20 or more years. The frontal scalp was the most common location of alopecia; the parietal scalp and the entire scalp with diffuse hair loss were also frequent sites. Prior to biopsy, female pattern alopecia was the most common clinical diagnosis; lichen planopilaris and scarring alopecia were also frequent diagnoses. After the biopsy, pseudopelade was the most common diagnosis; lichen planopilaris and female pattern alopecia were also frequently observed. The pathogenesis of incidental syringomas and syringomatous proliferation in areas of scalp hair loss is postulated to be secondary to subclinical alopecia-related reactive changes.

## Introduction

Syringomas are benign neoplasms of eccrine sweat ducts. Syringomas usually present as 1 to 4 millimeters, localized or generalized, flesh to slightly yellow-colored papules. They occur predominantly in the periorbital region of middle-aged women; the scalp is rarely affected [1].

Histologically, syringomas consist of two components. There is an epithelial proliferation of sweat ducts with characteristic comma-shaped nests; the dermal stroma adjacent to the tumor consists of dense fibrous tissue. The clear cell variant of syringoma is rare and develops as a result of glycogen accumulation secondary to phosphorylase deficiency in the tumor [2].

Syringomas have been observed in association with various skin conditions. In addition, cutaneous disorders with alterations of the eccrine sweat ducts or syringomatous proliferations have also been described. In this latter setting, the eccrine lesions have been noted to be either focal and irregular or multifocal ductal dilatations or both without stromal components [3,4].

Syringomas on the scalp have been reported with alopecia (which is either scarring or non-scarring) as well as without hair loss. However, syringomatous proliferations have been more frequently observed with alopecias than syringoma [5-13]. We describe a woman with scarring alopecia caused by lichen planopilaris who had incidental clear cell syringomas in the biopsy site of her hair loss. We also review the characteristics of patients with alopecia-associated syringomas and syringomatous proliferations.

## Case presentation

A 71-year-old woman sought evaluation for her hair loss. Her scalp examination revealed a scarring alopecia. Lichen planopilaris was considered based on her clinical presentation.

**Figure 1:**
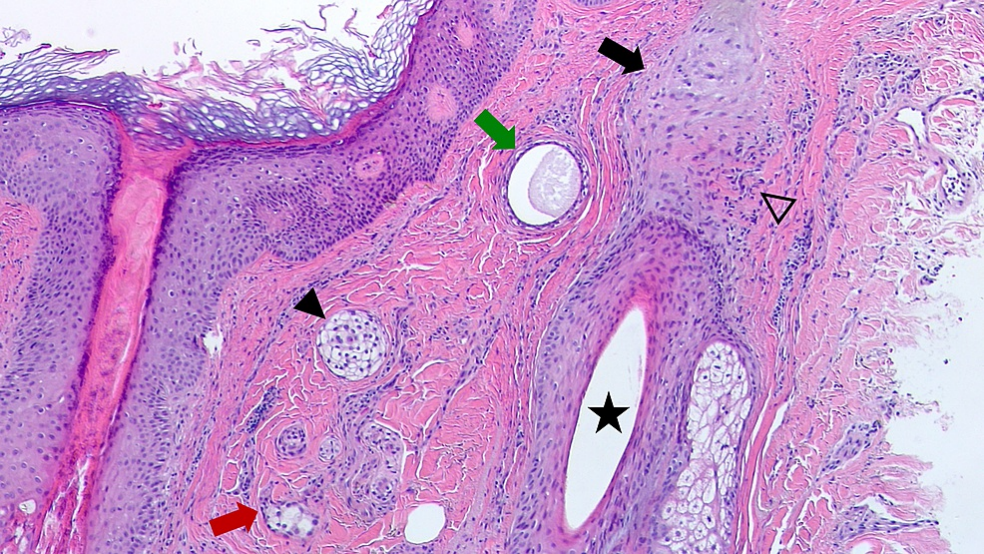
Clear cell syringoma and lichen planopilaris Low magnification shows a clear cell syringoma (solid black arrowhead); some of the syringoma have tails of epithelial cells (red arrow). A syringoma duct is also present (green arrow). In the adjacent dermis, there are features of lichen planopilaris: an inflamed hair follicle (black star), which has perifollicular fibrosis (black arrow) and chronic inflammation (open black arrowhead). (Hematoxylin and eosin, 100x).

**Figure 2:**
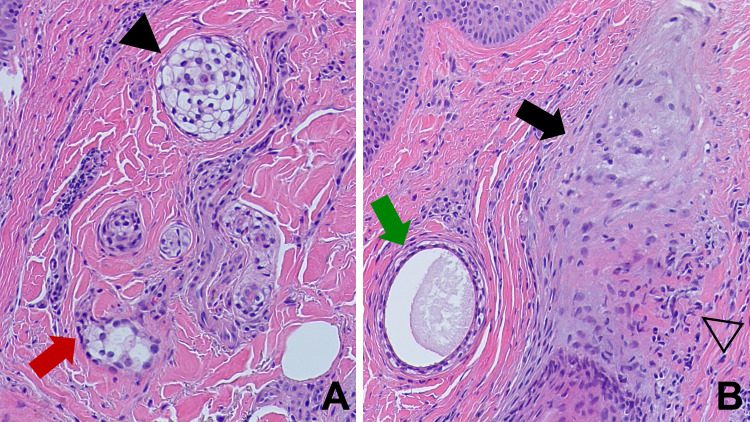
Scarring alopecia-associated clear cell syringoma Higher magnification demonstrates the clear cytoplasm of the syringoma cells (solid black arrowhead) and that some of the ducts show comma-like tails of epithelial cells (red arrow), making tadpole-like architectures (A). Perifollicular fibrosis (solid black arrow) with an accompanying lymphocytic infiltrate (open black arrowhead) and a syringoma duct (green arrow) are also present (B). (Hematoxylin and eosin: A, x200; B, x200).

**Figure 3:**
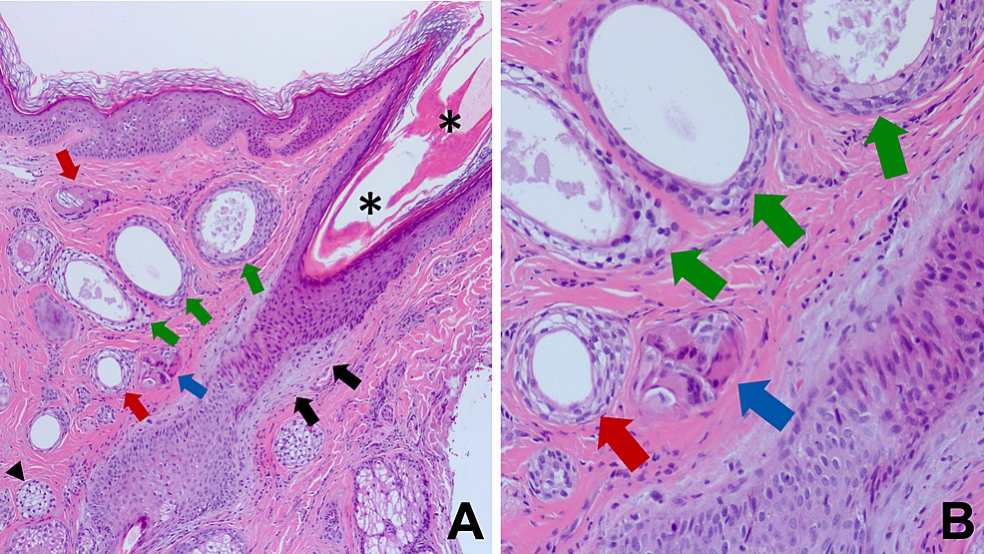
Lichen planopilaris-associated syringoma Low (A) and higher (B) magnification views of a dilated hair follicle infundibulum (black asterisks) with perifollicular fibrosis (solid black arrows) are adjacent to dilated syringoma ducts, some with clear cells (green arrows), and clear cell syringoma with (red arrows) and without (solid black arrowhead) comma-like epithelial cell tails; a small foreign body granuloma (blue arrow) is also present (A). These findings can also be observed at higher magnification (B). (Hematoxylin and eosin: A, x100; B, x200).

A 3-millimeter punch biopsy was performed from an area of hair loss on her scalp. Microscopic evaluation of the tissue biopsy showed fibrosis and lymphocytic infiltration around the isthmus of the hair follicles. These findings confirmed the clinically suspected diagnosis of lichen planopilaris (Figures 1-3).

In addition, in the superficial and mid dermis, embedded in the dense collagenous stroma and located close to an affected follicle, there were enlarged eccrine sweat ducts and cell islands that were irregular in shape and size; these findings established a concurrent diagnosis of eccrine syringoma. The benign syringoma tumor islands and eccrine ductal structures had more than two cell layers; some eccrine tumors were entirely composed of cells with clear cytoplasm, representing the clear cell variant of syringoma. More than half of the ducts appeared solid and many had a comma-like tail. Some of the ducts appeared both solid and cystic because of the increased epithelial linings. Secretory material was mostly present in the lumen of the enlarged ducts (Figures 1-3).

The syringoma cells were relatively uniform, with a clear or a finely granular eosinophilic cytoplasm. Nuclei were eccentric, small, and dark with a dense chromatin pattern and an indistinct nucleolus. Atypia was not evident and no mitotic figures were observed. There was no necrosis in the tumor and no invasion of either deeper or neural structures (Figures 1-3).

Several foreign body type multinucleated giant cells were also present in the dermis. There was no birefringent material observed under polarized light; hence, excluding the diagnosis of keratin granulomas. Therefore, it is possible that the granulomatous inflammation was caused by ruptured ducts near the epidermis (Figures 3A, 3B).

## Discussion

There are four clinical variants of syringomas: localized, disseminated, associated with Down syndrome, and inherited forms. Syringomas are derived from the lowermost intraglandular eccrine sweat gland. Although a syringoma occasionally appears as a solitary lesion, they usually present as multiple tumors [14,15].

Histologically, syringomas have numerous small eccrine ducts that are surrounded by fibrous stroma. The ducts are usually lined by two rows of epithelial cells, of which the inner row may appear vacuolated. Some of the ducts possess small, comma-like tails of epithelial cells; therefore, the lesions have been described to have a “tadpole” appearance. Solid strands of basophilic epithelial cells - independent of the ducts - are also seen in syringoma. Keratin cysts, that resemble milia, may also be present; sometimes they rupture and produce a foreign-body reaction [14].

Immunohistochemistry staining can be helpful, but are usually not necessary, to confirm the diagnosis of a syringoma. Carcinoembryonic antigen (CEA), cytokeratin 5, cytokeratin AE1/AE3 and epithelial membrane antigen (EMA) demonstrate positive staining in syringomas. The pathologic differential diagnoses of syringoma include desmoplastic trichoepithelioma, microcystic adnexal carcinoma (MAC), morpheaform basal cell carcinoma, and syringoid carcinoma [12,16,17].

The clear cell syringoma, which results from glycogen accumulation, is a rare histologic variant of this tumor. Clear cell syringomas are clinically indistinguishable from ordinary syringomas. Clear cell variant syringomas have been strongly associated with diabetes mellitus, in which phosphorylase deficiency occurs [2].

Although clear cell syringoma is considered to be uncommon, a study of 34 cases in India and a study of 244 cases in France both showed much higher frequencies of clear cell variant (85.3% and 8.5%, respectively). In addition, both studies revealed no correlation between the observation of clear cell syringoma and diabetes [1,16].

Histologically, the clear cell variant syringoma predominantly has cell islands that are irregular in size and shape with only a few ducts and strands. The tumor islands are usually composed entirely of clear cells. In addition, they have more cell layers than are generally seen in ordinary syringomas [12].

Incidental, also referred to as subclinical, syringoma and syringomatous proliferation have been observed microscopically in biopsy specimens from several dermatologic conditions. The differentiation between syringoma and syringomatous proliferation can be challenging; syringomas are histologically different from syringomatous changes by the presence of a dense collagenous background and the proliferation of epithelial nests with tadpole appearance. Eccrine sweat duct alteration, or syringomatous proliferation, has been postulated to be a reactive change to either acute inflammation, dermal fibrosis, or mechanical compression with possible occlusion of the acrosyringium [3,4,10].

Patterns of eccrine sweat gland proliferation were evaluated in two studies. Mehregan, in 1981, observed 48 specimens that showed eccrine sweat duct proliferation, from a collection of 40,000 routine biopsy specimens. Subsequently, in 1988, Luther and Altmeyer noted reactive processes of eccrine sweat glands in the 20,000 routine biopsy specimens they reviewed; however, the investigators did not specify the number of syringomatous proliferation they observed [3,4].

Syringomatous changes observed by not only Mehregan but also Luther and Altmeyer included cystic duct dilation, proliferation of ductal epithelium (such as small cell buds and “rope ladder-like” papillomatous projection into the cavity), and retention of secretory material. Mehregan also noted aggregates and nests of squamous metaplasia and branching ducts. In addition, Luther and Altmeyer described reactive changes of the secretory segment and periglandular connective tissue of eccrine gland [3,4].

Coexisting syringomas have been reported in various benign (intradermal nevus) and malignant (basal cell carcinoma) skin conditions [17,18]. In addition, syringomas were observed in scarring [5,8] and non-scarring alopecias [7,9,11]. Similarly, syringomatous changes have also been noted in both scarring and non-scarring alopecias [6,10,12,13]. Furthermore, syringomatous changes have been described in benign and malignant neoplasms (such as melanocytic nevus, basal cell carcinoma, cutaneous T-cell lymphoma, and keratoacanthoma), drug reactions (such as reactions to chloride hexahydrate and tetrachlorodibenzo-p-dioxin), and inflammatory dermatoses (such as eczema and prurigo nodularis) [19].

Lichen planopilaris is a variant of lichen planus, which predominantly affects the scalp. Clinically, Lichen planopilaris may initially present with perifollicular erythema and scaling; after loss of the hair follicles, the scalp is smooth and shiny. Lichen planopilaris is classified as a primary lymphocytic cicatricial alopecia [20].

Histologic findings of lichen planopilaris are variable and depend on the degree of disease progression. In early lesions, there is a vacuolar interface change with moderately dense perifollicular lichenoid lymphocytic cells infiltrating the hair follicle at the level of the infundibulum and isthmus. In more developed lesions, concentric lamellar perifollicular fibrosis occurs, and the lichenoid infiltrates "backs away" from the follicle; mucinous perifollicular fibroplasia with an absence of the interfollicular dermal mucin in the upper dermis has also been described. End-stage lichen planopilaris shows vertically oriented fibrotic tracts replacing the destroyed hair follicles with loss of elastic fibers [20]. In addition to our patient, syringoma and syringomatous proliferation have both previously been reported in patients with lichen planopilaris [8,12].

Alopecia-associated syringomas or syringomatous proliferation, including our patient, has been described in 17 individuals (Table 1) [5-13]. Sixteen of the patients (94%) were women. The age of the women ranged from 33 years to 83 years (median, 57 years). The man was 47 years old when diagnosed.

**Table 1:** Clinical and pathologic features of syringoma and syringomatous proliferation in patients with alopecia Abbreviation: AA: alopecia areata; app.: appearance; C: case; CD: cystically dilated; Clin: clinical; CR: current report; Df: diffuse; DFT: dense fibrous tissue; DLE: discoid lupus erythematosus; Dur: duration; Dx: Diagnosis; ESD: eccrine sweat duct; FPA: female pattern alopecia; Fr: frontal; GS: glandular structure; HF: hair follicles; HL: hair loss; LPP: lichen planopilaris; M: man; NOS: not otherwise specified; NS: not stated; NSA: non-scarring alopecia; Oc: occipital; Pa: parietal; Path: pathologic; PP: pseudopelade; Ref: reference; SA: scarring alopecia; SP: syringomatous proliferation; SYR: syringoma; TA: traction alopecia; TH: thinning of hair; TPA: tadpole appearance; Vr: vertex; W: woman; w/: with; y: years; 2º: secondary; ≥: greater than or equal to; &: and *Bilateral lesions

C	Age Sex	Site; Dur	Morphology	Clin Dx	Microscopic description	Path Dx	Ref
1	47y M	NS; 20y	Patchy SA w/ erythema & micro-nodular appearance	SA (DLE)	Cystic structures w/ thick and irregular elastic fibers and hyalinized collagen	SA (NOS) 2º to SYR	[5]
2	33y W	Oc; NS	Incomplete HL	SA (NOS)	≥ five CD ESD	SA (PP) w/ SP	[6] C2
3	43y W	Fr, Pa; NS	Incomplete HL	NSA (FPA)	≥ five CD ESD	NSA (FPA) w/ SP	[6] C4
4	50y W	NS; NS	Patchy complete HL	NSA (AA)	≥ five CD ESD	NSA (AA) w/ SP	[6] C7
5	50y W	Df; 20y	TH	NSA (FPA)	GS w/ a double layer, sometimes w/ TPA, embedded in DFS; GS impinged upon but preserved adjacent adnexa	NSA (FPA) 2º to SYR	[7]
6	53y W	NS; 3y	Patchy incomplete HL	TA	≥ five CD ESD	SA (PP) w/ SP	[6] C8
7	56y W	Fr*, Pa*; 0.5y	TH w/ loss of HF	SA (LPP)	Double-layered ESD, some w/ TPA, in a DFT	SA (LPP) w/ SYR	[8]
8	57y W	Df; 22y	TH w/ loss of HF	NSA (NOS)	Scattered cystic growth surrounded by DFT with loss of elastic fibers and follicular atrophy	NSA (NOS) 2º to SYR	[9]
9	57y W	NS; NS	Progressive SA	SA (NOS)	Sweat gland cyst formation which resembles syringoma	SA (NOS) w/ SP	[10]
10	58y W	Df; 5y	TH w/ loss of HF	NSA (NOS)	Dilated ESD, many with TPA, but no DFT	NSA (NOS) w/ SYR	[11]
11	60y W	Vr; NS	Extensive HL	NSA (FPA)	≥ five CD ESD	SA (PP) w/ SP	[6] C6
12	62y W	NS; 20y	Patchy incomplete HL	SA (NOS)	≥ five CD ESD	SA (PP) w/ SP	[6] C1
13	63y W	Vr; NS	HL	NSA (FPA)	≥ five CD ESD	NSA (FPA) w/ SP	[6] C5
14	67y W	NS; NS	A plaque of HL	SA (DLE)	≥ five CD ESD	SA (PP) w/ SP	[6] C3
15	67y W	NS; NS	Severe SA	SA (LPP)	NS	SA (LPP) w/ SP	[12]
16	71y W	Fr; NS	Alopecia	SA (LPP)	Cystic & solid ESD consisting of ≥ two layers mostly comprised of clear cells, w/ TPA; independent strands of epithelial cells in a DFT	SA (LPP) w/ SYR	CR
17	83y W	Fr, Pa*; 2-3y	Well-defined patchy complete alopecia	NSA (AA)	Dilated ESD of varying size and shape with occasional solid lobules, only minimal focal fibrosis	NSA (AA) w/ SP	[13]

The durations of alopecia ranged from six months to 22 years in the reported eight patients. The median duration was five years in women (seven patients); almost half of the women had a duration of alopecia of more than 20 years. The alopecia had been present for 20 years in the man.

One or more sites of alopecia were present. The most common scalp location of alopecia was the frontal area (four patients), followed by either the parietal area or the entire scalp demonstrating diffuse hair loss (three patients each). Other sites included the vertex (two patients), and the occipital area (one patient) of the scalp.

Various clinical diagnoses, prior to biopsy, were suggested by the patient’s physicians. The most common diagnoses were female pattern alopecia (four patients), lichen planopilaris (three patients) and scarring alopecia (not otherwise specified) (three patients). Other diagnoses included alopecia areata (two patients), discoid lupus erythematosus (two patients), nonscarring alopecia (not otherwise specified) (two patients), and traction alopecia (one patient).

The biopsy-established diagnoses of alopecia most frequently included pseudopelade (five patients), lichen planopilaris (three patients) and female pattern alopecia (three patients). Other diagnoses were alopecia areata (two patients), scarring alopecia (not otherwise specified) (two patients), and non-scarring alopecia (not otherwise specified) (two patients). Hair loss in three of the patients - one with scarring and two with non-scarring alopecia - was speculated to have resulted from the syringoma [5,7,9].

Indeed, our patient’s subclinical syringoma was an unanticipated pathologic change in the setting of lichen planopilaris. The microscopic findings not only demonstrated features of lichen planopilaris, but also those of syringoma: dense collagenous fibers, solid strands of epithelial cells, and frequent tadpole appearances of the ducts and nests without infiltrative growth into perineural or deeper structures. In addition to those findings, clear cytoplasm was observed in most of the eccrine cells establishing a diagnosis of a clear cell syringoma. We postulate that the finding of an eccrine tumor in our patient’s skin biopsy represented the coincidental association of a clear cell syringoma in a patient with lichen planopilaris.

## Conclusions

The observation of syringoma, a benign eccrine tumor, or syringomatous proliferation and alopecia has been described - including the woman in this report - in 17 patients: one man and 16 women. The reported patient had lichen planopilaris-related hair loss; the subclinical clear cell variant of syringoma that was observed in the biopsy specimen of her alopecia was an incidental finding. Although syringomas have been postulated as the cause of hair loss in some of these individuals, alopecia-associated reactive changes are hypothesized as the etiology of the sweat duct proliferation for most of the hair loss patients in whom this unique phenomenon has been observed.
